# Subcapital Realignment of the Displaced Pediatric Radial Head—Technical Description and Early Clinical Results

**DOI:** 10.3390/children13081026

**Published:** 2026-08-01

**Authors:** Daniel J. Epstein, Jitendra Balakumar, Erich Rutz, Kemble K. Wang

**Affiliations:** 1 Department of Orthopaedics, Royal Children’s Hospital, Melbourne, VIC 3052, Australia; jit.balakumar@rch.org.au (J.B.); erich.rutz@rch.org.au (E.R.); kemble.wang@rch.org.au (K.K.W.); 2 Department of Orthopaedics, Western Health, Melbourne, VIC 3011, Australia; 3 Clinical Sciences, Murdoch Children’s Research Institute, Melbourne, VIC 3052, Australia; 4 Department of Paediatrics, University of Melbourne, Parkville, VIC 3010, Australia; 5 Department of Orthopaedics, Eastern Health Box Hill Hospital, Melbourne, VIC 3128, Australia; 6 Department of Surgery, Monash University, Clayton, VIC 3800, Australia

**Keywords:** pediatric trauma, fracture, radial neck, subcapital realignment, surgical technique, trauma

## Abstract

**Highlights:**

**What are the main findings?**

- A technical note is presented describing a modified open reduction technique for pediatric radial neck fractures that aims to preserve the periosteal blood supply.
- Early clinical experience of a 15-patient series shows a 100% union rate with low rates of growth arrest and avascular necrosis in early results, but longer follow-up is needed.

**What is the implication of the main finding?**

- This technique shows promise, but longer-term follow-up and comparative studies are needed to definitively conclude any advantage over traditional methods of open reduction for pediatric radial neck fractures.

**Abstract:**

**Background:** Displaced pediatric radial neck fractures are challenging injuries. Adequate reduction can be difficult to achieve via closed methods. Open reduction has been associated with avascular necrosis and growth arrest due to soft tissue stripping during surgery. We describe a modified technique that adopts lessons learned from subcapital realignment procedures of the proximal femur, prioritising preservation of the periosteal sleeve with the aim of reducing complications associated with open reduction. **Methods:** The modified technique includes visualisation of the radial neck fracture through the torn periosteum, preserving intact periosteum, limited shortening of the diaphyseal fracture site, followed by open reduction and fixation with retrograde elastic intramedullary nailing. A retrospective chart review of 15 patients treated with this technique was undertaken to report early clinical and radiographic outcomes. **Results:** Mean age at injury was 7.7 years (range 4–11). Mean follow-up was 9.9 months (range 4.5 to 20 months). There was 100% fracture union achieved at mean 9.3 weeks (SD 4.9) post-surgery. Mean flexion was 139° (SD 9°), extension 1° (SD 7°), supination 66° (SD 23°) and pronation 79° (SD 21°). There were no cases of postoperative radial head deformity or instability. One patient showed radiological signs of avascular necrosis. Six of 15 patients experienced at least one complication, including heterotopic ossification, stiffness requiring release, physeal closure and nail migration requiring revision. **Conclusions:** We describe a technique that aims to preserve periosteal blood supply during open reduction and fixation of pediatric radial neck fractures. Early clinical experience suggests promising results, but longer-term follow-up is required before any advantage over existing open reduction techniques can be concluded.

## 1. Introduction

Radial head and neck fractures in the pediatric population account for up to 14% of all pediatric elbow fractures [1,2], with the most common injury being to the radial neck [1,3,4] following a fall on an outstretched hand [3]. As the pediatric radial head is largely cartilaginous, traumatic force across the elbow gets transmitted to the relatively weak proximal radius physis [3] where injury can result in Salter–Harris type I and II fractures. When treating displaced fractures, a closed reduction to restore alignment is preferred but can be challenging for markedly displaced fracture patterns or delayed presentations. On the other hand, accepting suboptimal reduction has morphological and functional impacts. Suboptimal reduction can lead to radiocapitellar joint subluxation, dislocation, loss of range of motion, and late degenerative changes [5]. The functional outcomes for these fractures in children are considerably worse than similar injuries in adults [3,5].

As a result, open reduction is sometimes required for displaced radial neck fractures—for those that cannot be adequately closed-reduced, as well as late presenting fractures or revision procedures [3,6]. Traditionally, the main concern with performing open reduction is an increased risk of avascular necrosis (AVN) to the radial head [1,3,7], with some studies reporting a risk of AVN up to 50% [8]. This is thought to be caused by soft tissue stripping and periosteal peeling from the fracture site during surgery with subsequent injury to the vascular supply to the radial head [9]. As a result, both AVN of the radial head and growth arrest are concerns when treating these injuries and can lead to cubitus valgus deformity, periarticular ossification, proximal radius overgrowth, chondrolysis and secondary arthritis [3,7]. Further, complex injuries that necessitate more invasive treatment or where inadequate reduction has been accepted are associated with higher complication rates and worse outcomes [3,10].

**Figure 1:**
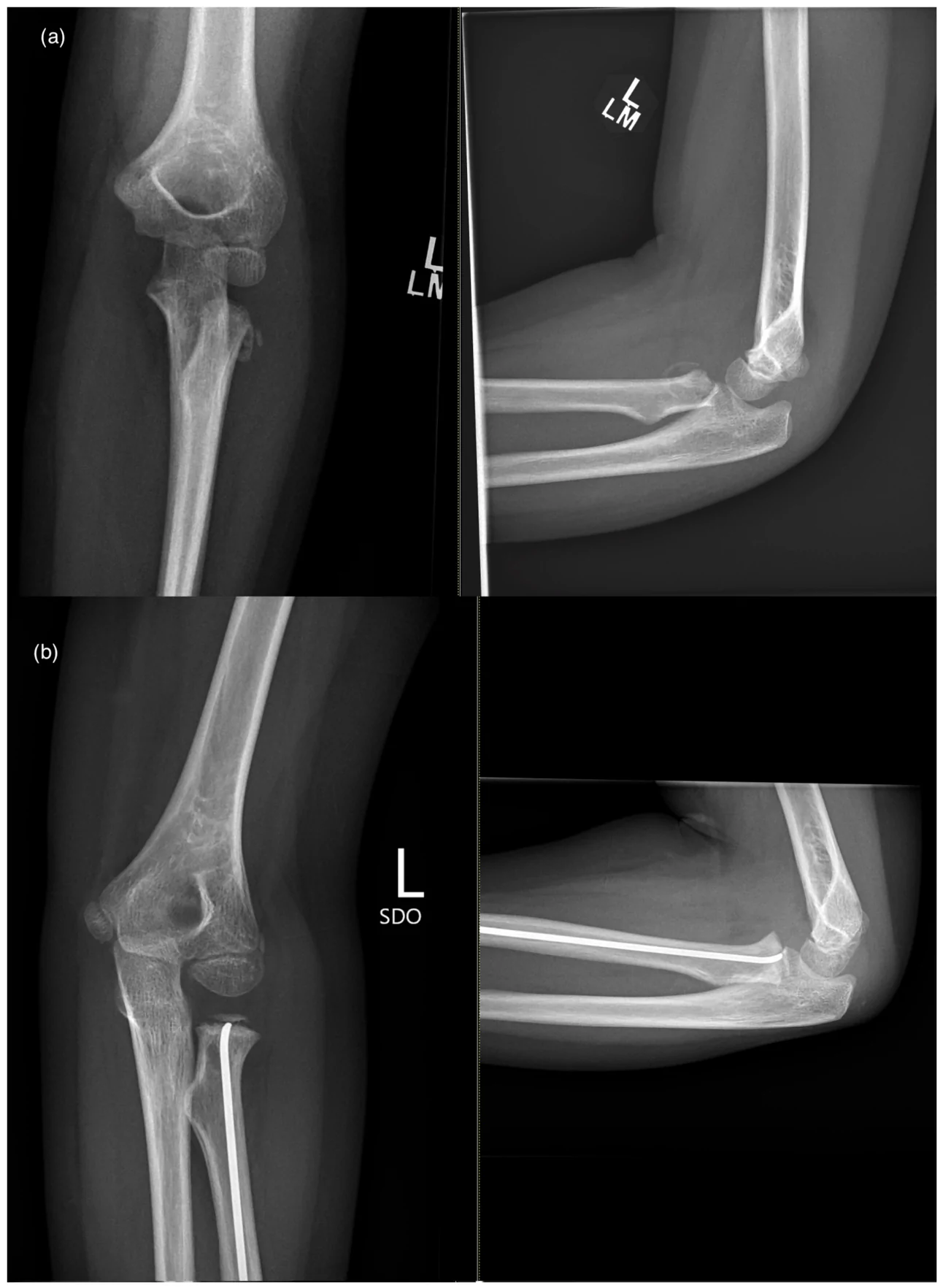
Radiographs depicting a displaced radial neck fracture; (**a**) preoperative radiographs; and (**b**) postoperative radiographs depicting a united fracture following the subcapital realignment procedure.

Since 2022, a modification of the traditional open reduction technique for radial neck fractures has been routinely performed at our institution that aims to reduce the risk of these complications (Figure 1). This technique adopts lessons learned from subcapital realignment procedures of the proximal femur for slipped upper femoral epiphysis conditions. It prioritises adequate exposure, anatomic reduction and preservation of the periosteal sleeve with the aim of preserving the periosteal blood supply. Henceforth in this manuscript, this technique will be referred to as subcapital realignment of the radial head.

**Figure 2:**
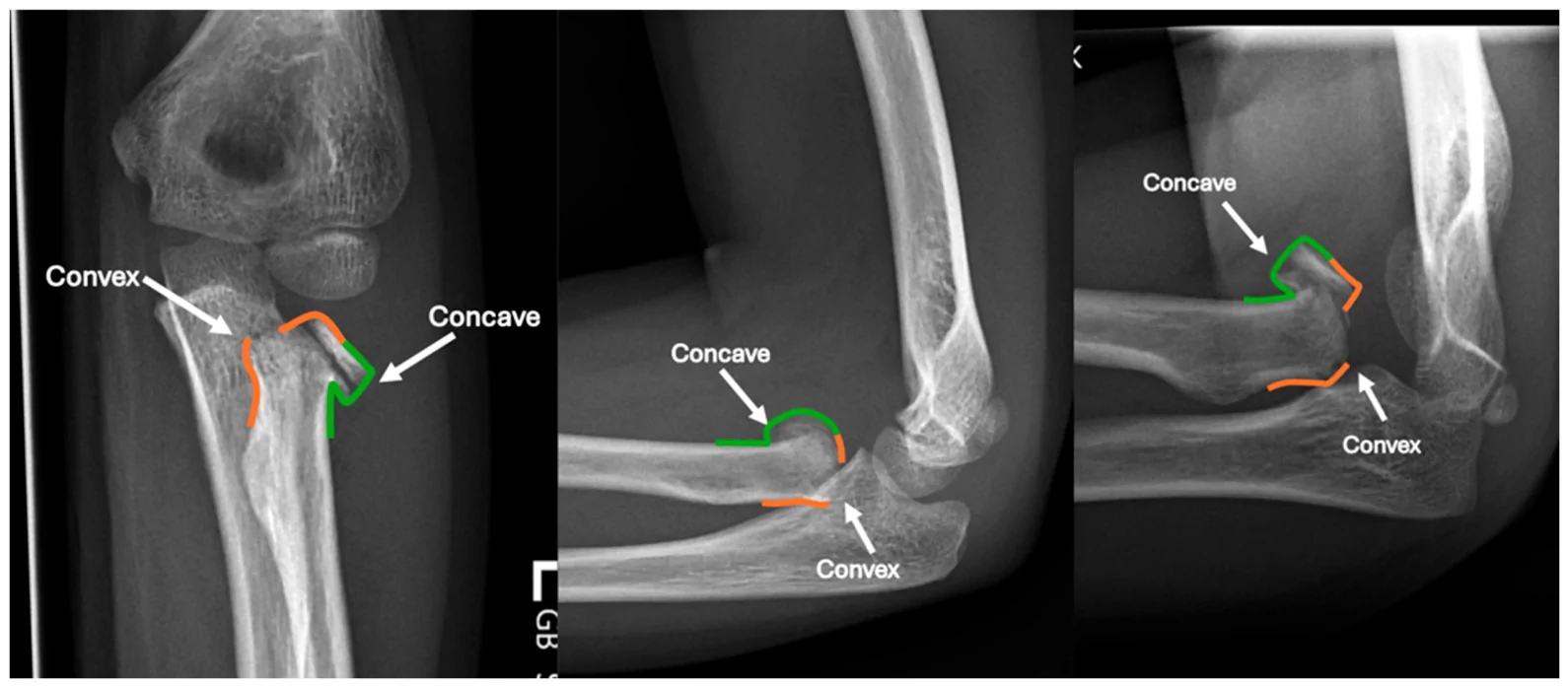
Annotated radiographs demonstrating the concave and convex sides of the radial neck fracture, with the intact periosteum expected on the concave fracture side (green) and the torn periosteum expected on the convex side (orange).

An important concept when aiming to preserve the periosteal blood supply is understanding the direction of fracture displacement and the result on the periosteum. In radial neck fractures, the radial head will often displace towards one side of the radial shaft—this de-tensions the intact periosteum and creates a “concave” periosteum in this direction (Figure 2). Similarly, the radial head has displaced away from the opposite side of the radial shaft which invariably tears the periosteum—creating a “convex” deformity at this location. The torn periosteum on the convex side usually extends approximately 25% of the circumference of the radial neck. The convex side is often located posteriorly on the radial shaft, and can be best appreciated in maximum forearm pronation. The fracture should be visualised through the periosteal defect on the convex side, while the intact periosteum on the concave side (anteromedial) must be meticulously preserved to minimise disruption to the blood supply of the radial head.

Importantly, open reduction of pediatric radial neck fractures should be reserved as a last resort. All fractures should first try to achieve a satisfactory reduction using closed methods, and the majority of fractures can be successfully treated in this manner. Achieving satisfactory closed reduction can be difficult with delayed presentations beyond 7 days post-injury.

**Figure 3:**
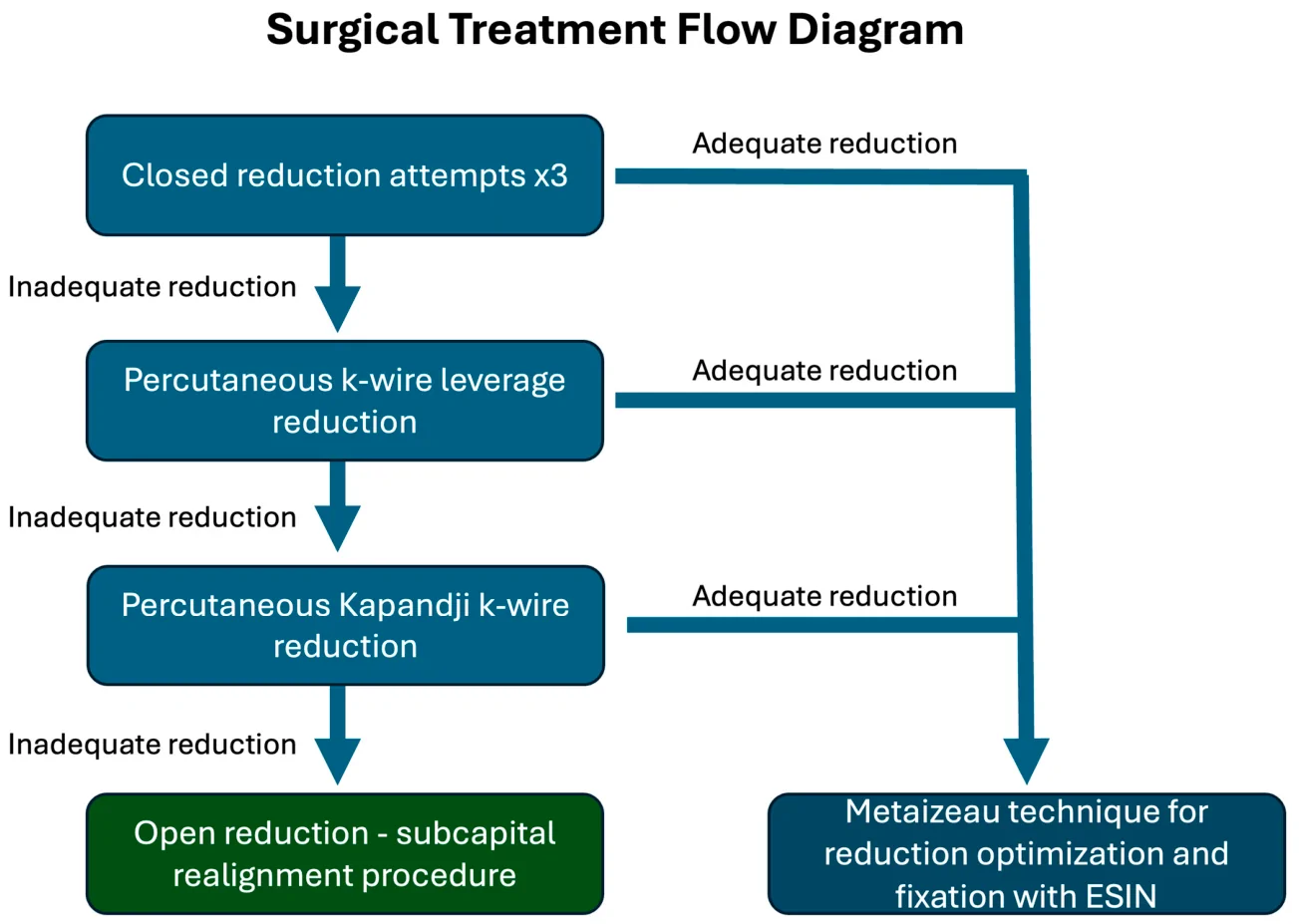
Flow diagram depicting surgical treatment decision making. All efforts are made to obtain an adequate reduction via closed methods before proceeding to an open reduction.

The senior author on this paper has previously published on a closed reduction technique involving varus stress, thumb pressure over the radial head then elbow flexion and forearm pronation [11,12]. Our treatment protocol involves three closed reduction attempts using this technique. If inadequate reduction remains, then percutaneous k-wire-assisted reduction is performed using either k-wire leverage or Kapandji reduction techniques as appropriate. Adequate reduction is correction in both supination and pronation to less than 15° of residual fracture angulation and less than 15% residual fracture translation with a congruent radiocapitellar joint. While target values for adequate reduction are debated in the literature, these limits have been chosen at our institution as some reports suggest remodelling deformity greater than this may be limited [8,13]. Multiple intramedullary passes using a Metaizeau reduction technique are deliberately avoided because repeated instrumentation of the radial head may compromise fixation and potentially increase the risk of physeal injury. Fractures that meet our criteria for adequate reduction can undergo further reduction optimization and subsequent fixation using a Metaizeau technique without open reduction. However, if adequate reduction has not been achieved in both pronation and supination, or radiocapitellar joint incongruity is present, then the authors proceed to an open reduction using the subcapital realignment technique (Figure 3).

The key surgical steps during subcapital realignment of the radial head are:

- Placement of a 2.0 mm retrograde elastic stable intramedullary nail (ESIN) prior to any attempted open reduction, with the tip ending just before the fracture site.
- Lateral approach to the elbow through an EDC-split interval, taking care to avoid any iatrogenic damage to the periosteum.
  ○ If needed for exposure, safe release of the lateral collateral ligament using a posterior “peel” method that allows easy and effective repair.
- Rotation of the forearm until visualisation of the torn periosteum at the “convex” side of the fracture.
- Utilising the existing window of torn periosteum, gently exaggerating the deformity to access the fracture site for debridement.
- Limited shortening of the diaphyseal side of the fracture to effectively de-tension the intact periosteum on the “concave” side of the fracture with the goal of minimising vascular disruption.
- Careful open reduction and realignment of the radial head.
- Fracture fixation taking previously sited ESIN and passing it across the fracture site and into the epiphysis to achieve stable fixation.

The technique is described below together with the early clinical results of 15 elbows treated using this procedure.

## 2. Surgical Technique

**Indications**

The subcapital realignment technique is indicated for Salter–Harris I or II fractures of the proximal radius that **cannot be adequately reduced** using closed or percutaneous techniques and meet ANY of the following criteria:

- Initial fracture angulation > 30°;
- Initial > 30% translation of the radial head/epiphysis relative to the radial shaft;
- Radiocapitellar joint incongruity or instability in supination or pronation;
- Delayed presentation with early callus formation;
- Revision surgery with prior unsuccessful closed methods.

**Room set-up and positioning**

The patient is positioned supine on the operating table with the affected extremity placed on a radiolucent hand table. A high arm tourniquet is applied. The surgeon is at the head-side of the hand table and the assistant in the axilla, with the image intensifier positioned to enter in-line with the hand table (at 90° to the operating table).

**Initial radius preparation**

The first step is to pass a 2 mm elastic stable intramedullary nail (ESIN) retrograde along the radial shaft using a standard distal radius styloid metaphyseal entry point, with the tip of the nail ending just distal to the radial neck fracture site. This is done prior to any attempt at open reduction as the impaction required to introduce the ESIN can re-displace the fracture and risks further damage to the remaining periosteal sleeve. The remaining nail is then cut 5–7 cm from the skin to remove excess length that will hinder the remainder of the operation while allowing sufficient length for final nail impaction when required.

**Exposure for open reduction**

**Figure 4:**
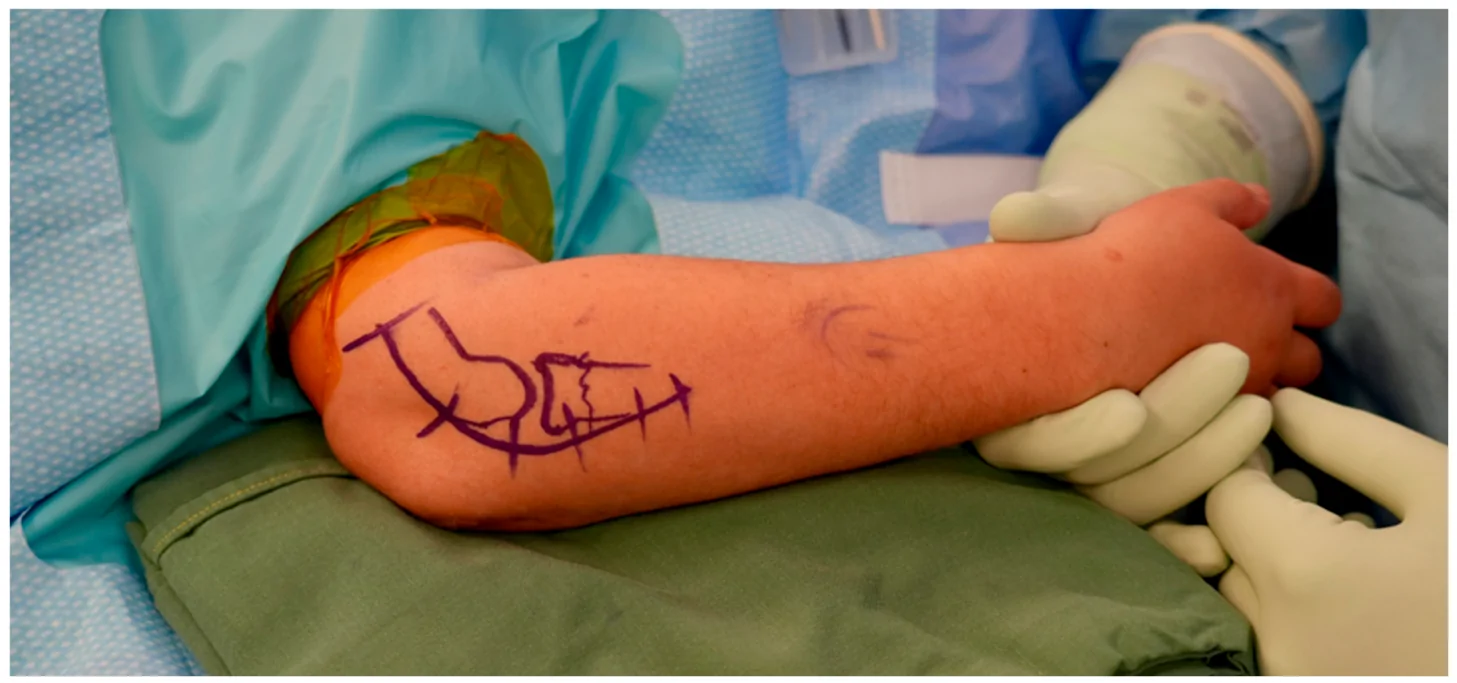
A curvilinear incision is made passing posterior to the radiocapitellar joint.

**Figure 5:**
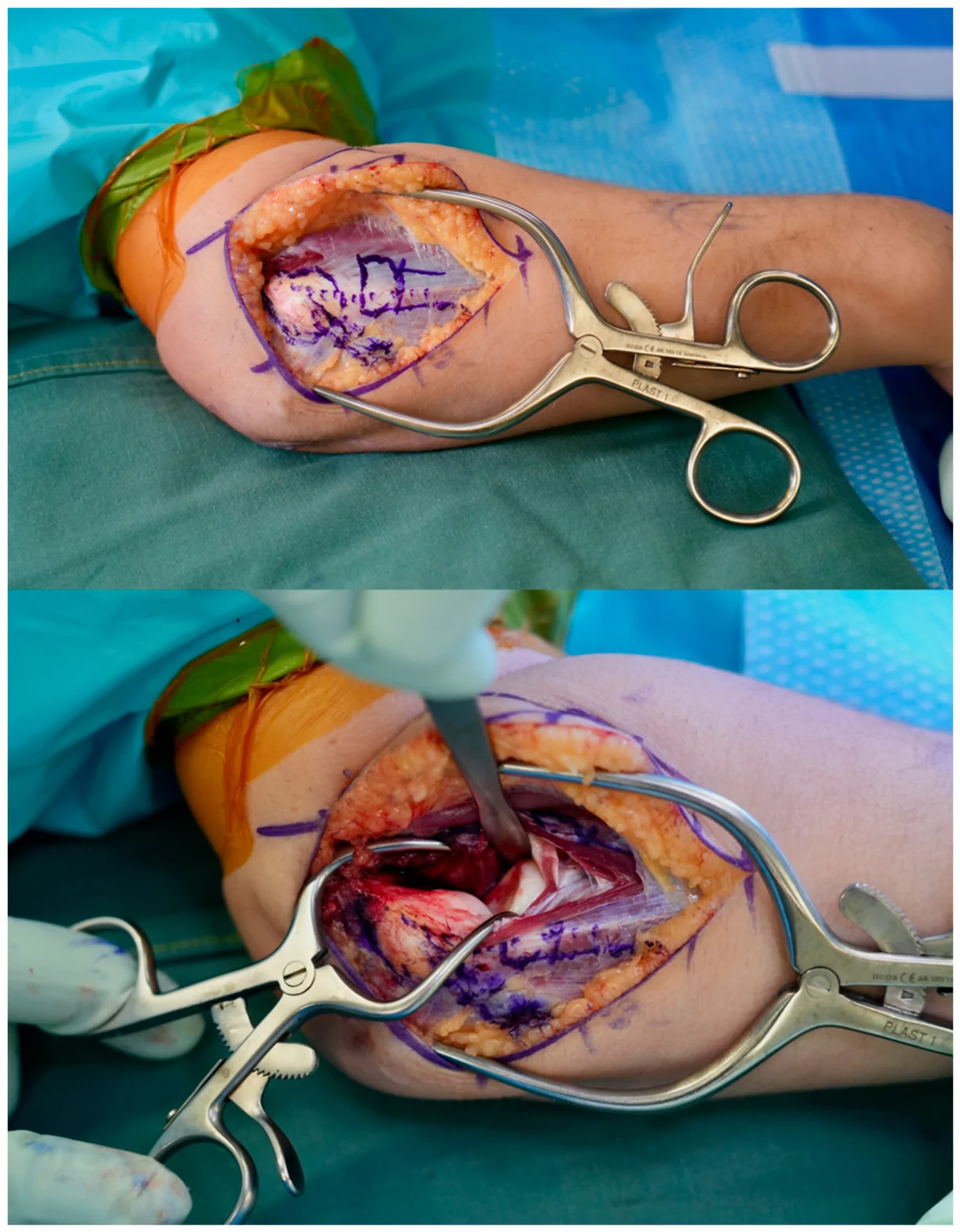
The radial head and neck are exposed using an EDC-split and lateral capsulotomy ensuring dissection remains anterior to the LUCL.

**Figure 6:**
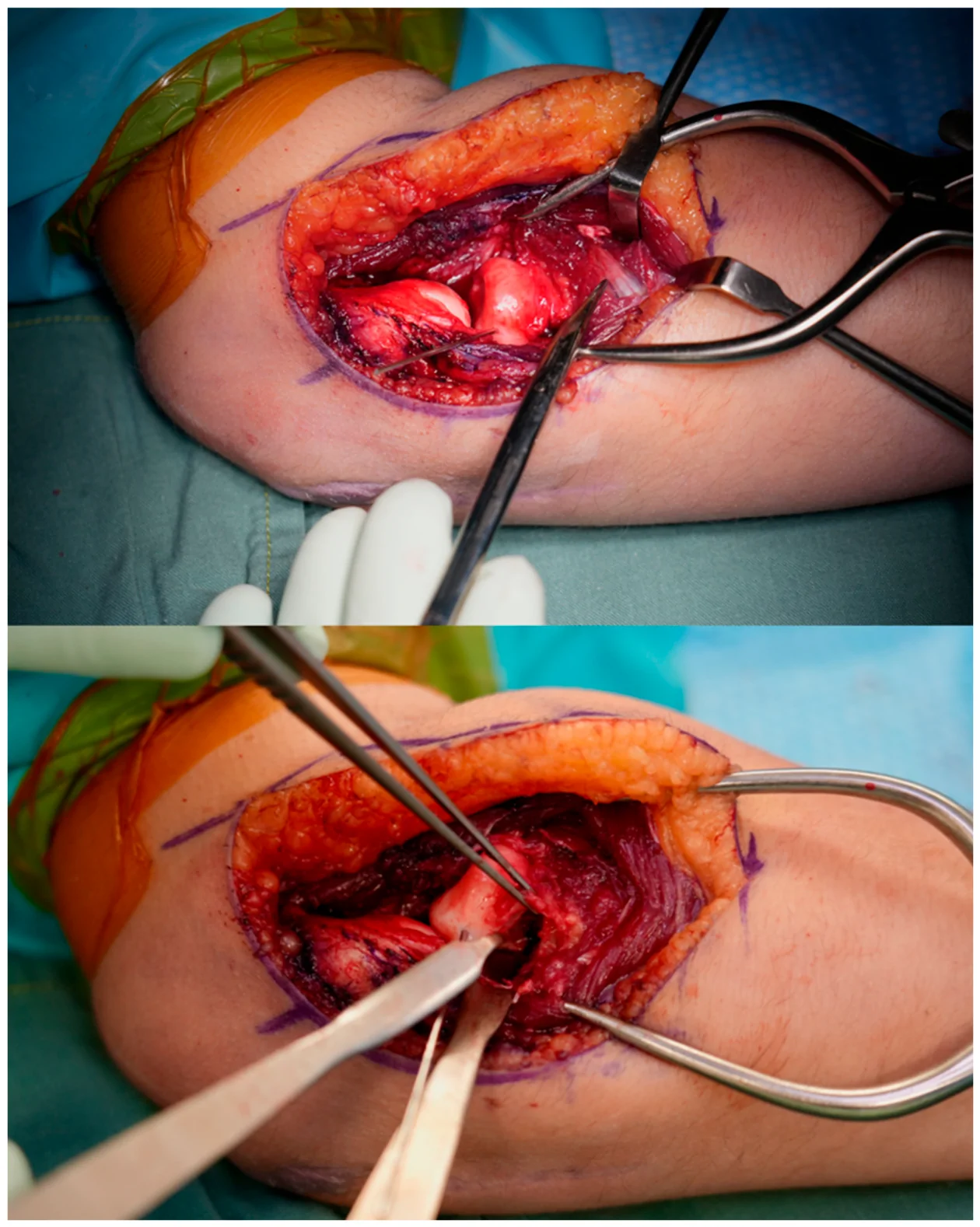
A 1.2 mm joystick wire is inserted into the radial epiphysis and the concave intact periosteum is identified and protected. Note—the PIN is identified in the top image and has been neurolysed and protected in this example.

With the forearm in pronation and the elbow flexed to 90 degrees, the lateral epicondyle and the radial head are identified (Figure 4). A curvilinear incision is made centred posterior to the radiocapitellar joint and full thickness flaps are developed to reveal the common extensor origin. Dissection is performed anterior to the lateral ulnar collateral ligament (LUCL) and starts at the lateral supracondylar ridge elevating extensor carpi radialis brevis (ECRB) and the anterior edge of extensor digitorum communis (EDC) from the distal humerus. It is important to stay anterior to the lateral epicondyle to protect the LUCL. The dissection and capsulotomy is then continued distally to access the radiocapitellar joint and radial neck, splitting the EDC tendon while keeping the forearm in pronation to protect the posterior interosseous nerve (PIN) (Figure 5). If distal radial neck exposure is required, the PIN can be neurolysed and protected (Figure 6). This involves incising the annular ligament for exposure and is safe to do—it does not carry any blood supply to the proximal radius. Care is taken to preserve the remaining periosteum on the radial neck, being particularly mindful of fragment displacement. At this point, the LUCL origin can be recessed at the lateral epicondyle with a peel in the posterior direction (rather than truncating the ligament) to allow greater access to the radiocapitellar joint if required.

**Fracture preparation and periosteal preservation**

Prior to exploration of the fracture site, it is critical to appreciate which side of the radial neck is the “concave” and which is the “convex” side of the fracture (Figure 1). As explained in detail above, the periosteum on the concave side is almost always intact while there is usually a tear in the periosteum on the convex side. The fracture should be visualised through this tear in the periosteum. Meticulous care is taken to preserve the intact periosteum on the concave side to minimise the chance of disrupting blood supply to the radial head.

**Figure 7:**
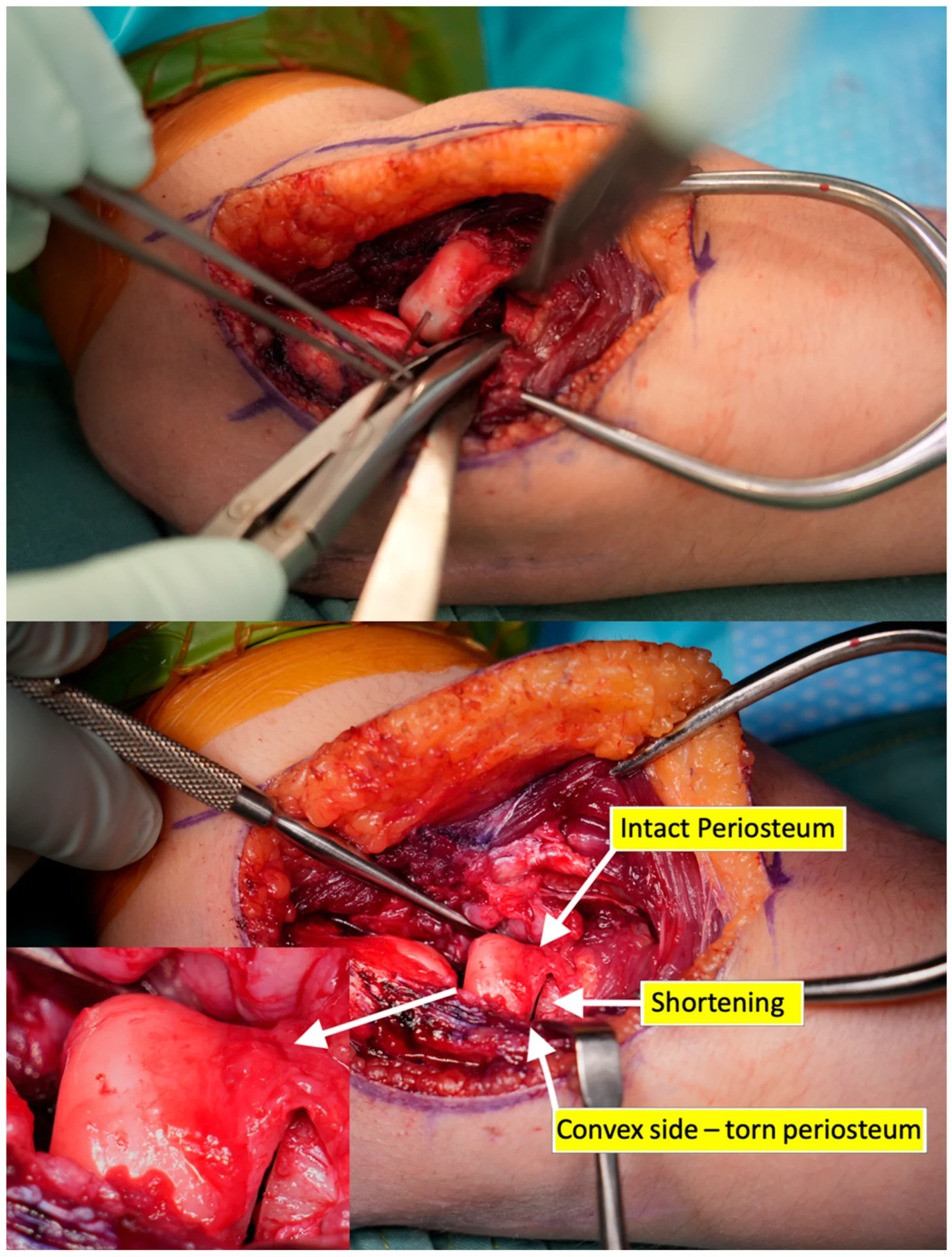
The radial head is displaced in the direction of the intact periosteum and the diaphyseal side of the fracture is shortened with a rongeur. This prevents tension on the preserved concave periosteum. The fracture is then reduced. Note—inset zoomed image of the radial head showing intact periosteum with periosteal blood vessels over the radial head and neck.

A unicortical 1.2 mm k-wire is then placed into the radial head as a joystick to control the proximal fragment (Figure 6). Using gentle digital pressure combined with joystick control, the radial head fragment is displaced further anteriorly in the direction of the intact periosteum to expose the diaphyseal side of the fracture. Haematoma, soft callus or comminution here is removed as necessary. Using a small rongeur, limited shortening (2–3 mm) of the diaphyseal side of the fracture is performed. The goal is to prevent any tension on the concave periosteum that could compromise blood supply upon fracture reduction (Figure 7)—a similar principle to callus removal and slight shortening of the femoral neck to prevent tension on the periosteal blood supply during subcapital realignment procedures of the hip. Slight shortening of the fracture also facilitates maintenance of reduction, as the intact periosteum can act as an unbalanced tension displacement force on the concave side of the fracture.

**Realignment and fixation**

**Figure 8:**
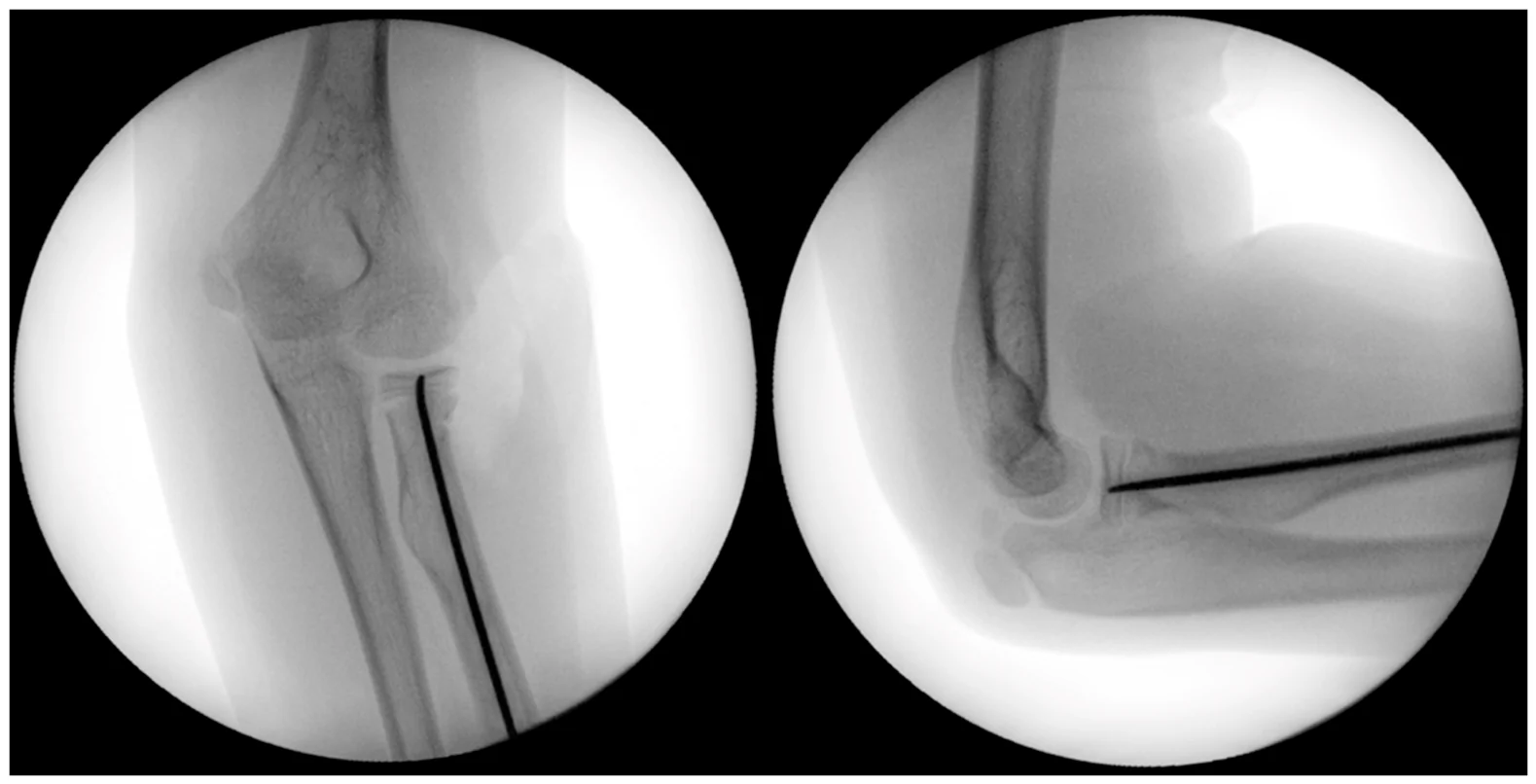
The 2 mm elastic nail is inserted retrograde along the radius to cross the fracture site and enter the ossification centre of the epiphysis to achieve fixation without breaching the articular surface of the radial head.

The radial head is then gently reduced back on to the shaft with digital pressure and joystick control. There should be a relatively equal overhang of the radial head on the neck in all directions to ensure the radial head is centred on the shaft, and the intact periosteum should remain tension free. Reduction is held with digital pressure while the elbow is then flexed to 90 degrees and the radial head is compressed concentrically against the capitellum with an axial load. This provides gentle compression and stability during impaction of the nail and prevents distraction and re-displacement at the fracture site. The previously positioned retrograde nail is then inserted further under image intensifier guidance to cross the fracture site, cross the physis, and finish within the ossified area of the epiphysis to achieve fixation (Figure 8). Rotation of the nail should be optimised prior to crossing the fracture site to achieve central purchase within the radial head. Care is taken to avoid breaching the articular surface and to avoid multiple passes into the epiphysis to prevent loss of fixation. The joystick k-wire is then removed and gentle pronosupination of the forearm is performed to confirm rotational stability of the fracture. The nail is now cut as short as possible at the distal radius insertion site and buried to prevent prominence. Any recession of the LUCL insertion is then repaired using an all-suture anchor.

**Arthrogram**

We routinely perform a post-reduction arthrogram for radiographic documentation and confirmation of reduction. An 18-gauge cannula is inserted percutaneously posterior to the incision, passing through anconeus and into the elbow joint under direct visualisation. The needle is removed but the plastic sheath left in situ. The capsule and muscle layer is then closed with a running 1 vicryl suture. Contrast dye is then injected into the cannula and image intensifier is used to confirm fracture reduction and radiocapitellar stability.

**Postoperative care**

The patient is splinted with an above-elbow backslab in neutral rotation for three weeks. At three weeks, full active and passive motion is allowed with a physiotherapist. Minimal loading on the affected limb is allowed until six weeks post-surgery. Resumption of sporting activities is permitted once radiological union is confirmed. The elastic nail is left in situ until fracture union is achieved and can then be removed on an elective basis.

## 3. Materials and Methods

A retrospective review was performed on all patients who underwent the subcapital realignment procedure of the radial head. All patients with a diagnosis of acute fracture and a minimum follow-up of six weeks post-surgery were included. Institutional ethics board approval was granted for this review. The aim of this review was to report our early clinical results in the context of the published literature on open reduction of pediatric radial neck fractures.

All medical records and relevant imaging were reviewed to assess patient demographics, mechanism and pattern of injury. Clinical and radiological outcome data were reviewed, including rates of complications such as avascular necrosis, growth arrest, radial head overgrowth, and further surgery. Radiological diagnosis of AVN was defined as the presence of abnormal sclerosis or lucency within the radial head on plain radiographs or CT imaging where available. Radiological diagnosis of growth arrest was defined as physeal closure earlier than expected relative to bone age. Measurements of fracture angulation and displacement relative to the radial shaft were performed on plain radiographs by the authors using previously published techniques [1].

Statistical analysis was performed using GraphPad Prism (version 11.0.2, GraphPad Software, San Diego, CA, USA (2026)). Continuous variables are presented as means ± standard deviation (SD) and were compared using the Mann–Whitney U test, while categorical variables were compared using Fisher’s exact test. All tests were two-tailed, with statistical significance defined as *p* < 0.05. Ninety-five percent confidence intervals (95% CIs) were calculated where appropriate.

This manuscript was prepared and reported in accordance with the Strengthening the Reporting of Observational Studies in Epidemiology (STROBE) statement and checklist [14]. The clinical photographs used in this manuscript were taken after written informed consent was obtained, and all images have been de-identified to maintain patient privacy.

## 4. Results

### 4.1. Demographics

We identified 17 patients with a Salter–Harris type I or II fracture of the radial neck treated with the subcapital realignment technique at our institution between 2022 and 2026 (Table 1). Two patients were excluded—one where surgery was a revision procedure for non-union 4 months post-injury, and one who was lost to follow-up before 6 weeks. The remaining 15 patients treated for acute fracture had a mean follow-up of 9.9 months (SD 4.8, range 4.5–20), with median follow-up being 8.6 months. The mean age at injury was 7.7 years (SD 2.1, range 4–11), with 9 of the 15 patients being female. Mean preoperative fracture angulation relative to the axis of the intact radial neck was 44° (SD 21°, range 18–92°) and mean translational displacement of the epiphysis relative to the intact radial neck was 46% (SD 18%, range 20–100%). Surgery occurred at a mean 6.1 days after injury (SD 4.1, range 1–14 days). Indications for open reduction were an incongruent radiocapitellar joint in two cases, and inadequate closed reduction in 13 cases with detailed reasoning listed in Table 2. Seven fractures were associated with additional injuries—two preoperative PIN palsies which both resolved following neurolysis at the time of fracture surgery, three ulna fractures which were fixed concurrently, one coronoid fracture and three instability injuries (two lateral ulnar collateral ligament repairs and one medial collateral ligament repair).

### 4.2. Outcomes

Fracture union was achieved in all patients (100%) at a mean of 9.3 weeks (SD 4.8, range 3.9–18.9) post-surgery (Table 3). At latest follow-up, range of motion recorded was mean flexion 139° (SD 9°, range 110°–145°), extension 1° (SD 7°, range −10°–20°), supination 66° (SD 23°, range 30°–90°) and pronation 79° (SD 21°, range 10°–90°). Only one patient had a pronosupination arc less than 100°. There were no cases of postoperative radial head subluxation, overgrowth or dislocation. One patient showed radiological signs of avascular necrosis for an overall AVN rate of 7% (95% CI 0–30%). Among the patients, 12 of the 15 underwent a second surgery for removal of the elastic nail at a mean of 5.4 months (SD 2.8, range 1.6–12.6) post-surgery. 

Descriptive mean range of motion values for isolated radial neck fractures and those with associated injuries are presented in Table 4; the small numbers in each subgroup preclude reliable conclusions regarding differences between groups. Patients with associated injuries tended to get surgery sooner compared with isolated injuries (4.6 vs. 7.5 days), and tended towards greater initial fracture displacement (54% vs. 38%).

### 4.3. Complications

Six patients experienced at least one complication in our early clinical experience. Four of these patients had isolated radial neck fractures.

Two patients had mild heterotopic ossification (HO) on their latest radiographs but were asymptomatic with full range of motion. One patient developed moderate heterotopic ossification with restricted supination to 30° and pronation to 10° at five months post-surgery—and is still undergoing physiotherapy at the time of writing. These three patients had isolated radial neck fractures. In each case, HO formation was located on the anteromedial ulna at the level of the radial neck fracture and likely represents a bone formation response to occult soft tissue injury at the time of fracture.

One patient had persistently limited supination to 10° due to scar tissue in the proximal radioulnar joint (PRUJ), and underwent arthroscopic PRUJ release at the time of elastic nail removal using a technique previously described by the senior author [15]. This improved supination to 80° at the time of surgery, and the patient has since maintained 45° of active supination. This patient had a preoperative PIN palsy that recovered by six weeks post-surgery, but this delay in active supinator function may have contributed to their limited rotational movement.

One patient had early proximal radius physeal closure at age 11 but remains asymptomatic at four months follow-up with no noticeable visual cubitus valgus.

One patient had proximal migration of the elastic nail through the radial head at eight weeks post-surgery and required nail removal and revision fixation with a 1.5 mm locking T-plate. They went on to achieve fracture union six weeks later, but latest follow-up at six months shows signs of AVN on CT imaging with multiple small areas of bone resorption not visible on plain radiographs. This patient has several risk factors for avascular necrosis with initial fracture displacement of 100% translation and 92° angulation, as well as undergoing revision surgery. They have minimal pain with a reduced flexion–extension arc of 20–110°, with pronation to 60° and supination to 40°.

## 5. Discussion

Displaced radial neck fractures are challenging to manage when closed reduction is unable to achieve acceptable alignment. We describe here a novel surgical technique for open reduction of radial neck fractures that utilises principles learned from subcapital realignment of the hip—optimizing surgical access, preserving the periosteum, minimising disruption to periosteal blood supply, and achieving anatomic reduction. To date, we have observed a 100% union rate with one case of AVN in early results.

Historically, complication rates following open reduction of radial neck fractures have been high [9], with reported AVN rates of up to 50% [8]. Falciglia et al. [7] reported on a cohort of 24 cases that underwent open reduction by an alternative technique without fixation for Salter–Harris type I and II radial neck fractures. They identified seven cases of premature physeal arrest resulting in deformity, and three cases of AVN—all of which were associated with poor outcomes. Similarly, Liu et al. [16] identified seven cases of premature physeal closure from a cohort of 24 similar fractures treated with open reduction. Our early clinical results have so far identified only one case of premature growth arrest and one case of AVN in a patient that required revision fixation surgery. However, our mean follow-up of 9.9 months is not adequate to fully understand the true rates of these complications following the subcapital realignment procedure.

Our early radiological results show promise with 100% fracture union in our series and no radiological deformity or malunion of the proximal radius. However, our functional outcomes can still be improved. Five of our fifteen patients achieved a supination range less than 60°. Postoperative rotational stiffness remains a concern and our results are consistent with other published series. This is an area for further optimisation with potential therapeutic targets including earlier mobilisation and more aggressive physical therapy. While the soft tissue dissection associated with this open reduction technique is more involved than mini-open or percutaneous methods, the authors hypothesise the advantages of identification and preservation of the intact periosteum, as well as anatomic fracture reduction, outweigh the disadvantages of a more invasive approach when closed reduction is unacceptable.

We propose that the use of an elastic nail over k-wire fixation offers several benefits. Elastic nails can be left in situ until fracture union is achieved—whereas k-wires need to be removed early to commence range of motion. Elastic nails also allow axial load sharing, with possible stability benefits over k-wires or headless screw fixation. Additionally, in the event of AVN and metalware cut-out, elastic nails are less detrimental to adjacent cartilage on the capitellum and proximal ulna when compared to headless screws. Elastic nails have also proven beneficial over fixation with k-wires in previous studies [9,16].

One patient identified in our initial cohort that was excluded from this series underwent the subcapital realignment technique in the setting of non-union. This patient underwent previous open reduction and k-wire fixation at an external institution which progressed to non-union, and subsequently underwent a subcapital realignment procedure at our institute four months after initial injury. While the approach and reduction technique remained unchanged from above, this patient underwent fixation with a 1.5 mm locking T-plate onto the proximal radius rather than an elastic nail due to poor bone quality found in the setting of non-union. They also had autologous bone graft inserted. The patient achieved radiographic union less than two months post-surgery with no complications, and was pain-free with full extension, flexion to 145°, supination to 80° and pronation to 70° at 12-month follow-up. Previous reports of radial neck fracture non-union treated with revision fixation have shown union with radiological abnormalities and motion restrictions [17]. While this is only a single case, the subcapital realignment technique may also have merit as a revision procedure and warrants further investigation in future studies.

### Limitations

Our early clinical experience report has several limitations. We present a small and heterogenous cohort that renders hypothesis testing limited to exploratory analysis only. Operative documentation did not consistently permit patient-level distinction between persistent incomplete reduction and apparent loss of reduction during pronation or supination. Our cohort also has inadequate follow-up duration to definitively report the rates of complication such as AVN or growth arrest. A larger sample size with longer-term follow-up is required to definitively report the rates of these complications using this technique. Future research will focus on longer-term follow-up and include a comparative cohort to allow for more detailed analysis of the safety and efficacy of this technique.

Further, we did not perform intraoperative blood flow assessments to confirm radial head perfusion post-reduction. Previous attempts at this by the authors have proven equivocal, as blood flow to the radial head in healthy cohorts is challenging to detect on perfusion monitoring compared with the hip where perfusion monitoring is more rewarding. Postoperative perfusion imaging was not performed, but this could be an area for future research to confirm blood supply to the radial head is preserved using this technique.

## 6. Conclusions

This technical note describes a reproducible technique for open reduction of displaced pediatric radial neck fractures with emphasis on how to anatomically reduce and hold the fracture while preserving the periosteum and minimizing disruption to the periosteal blood supply. Early clinical results suggest this technique may provide satisfactory short-term results, but comparative studies with longer-term follow-up are required before any advantages can be proven over traditional methods of open reduction for pediatric radial neck fractures.

## Figures and Tables

**Table 1:** Patient demographic information.

Patient	Age	Gender	Injury Mechanism	Days to Surgery	Initial Fracture Angle on XR	Initial Fracture Displacement on XR	Previous Procedures	Concomitant Injuries	Concomitant Procedures
1	8	F	Fall from sliding pole	1	40°	32%	-	-	-
2	5	M	Fall from scooter	10	41°	57%	Closed reduction in theatre	-	-
3	10	M	Fall from monkey bars	1	25°	20%	-	-	-
4	4	F	Knocked over by scooter	13	21°	41%	-	-	-
5	10	M	Fall from trampoline	6	45°	38%	Closed reduction in theatre with percutaneous k-wire fixation	-	-
6	6	F	Fall from bouncing castle	4	51°	51%	-	PIN palsy pre-op (delayed)	PIN neurolysis—recovered by 5/52
7	6	M	Fall from trampoline	3	63°	45%	-	Ulna fracture, PIN palsy pre-op	PIN neurolysis, pinning ulna. PIN recovered by 6/52
8	7	F	Fall from playground	4	47°	50%	-	Ulna fracture	Pinning of ulna
9	5	M	Fall from playground	9	18°	48%	Closed reduction in theatre	Ulna fracture	-
10	11	M	Fall from mountain bike	4	22°	52%	-	Coronoid fracture	Coronoid ORIF, MCL repair
11	10	F	Fall at gymnastics	1	92°	100%	-	Elbow dislocation	LUCL repair
12	6	F	Fall at gymnastics	7	37°	30%	-	LUCL avulsion	LUCL repair
13	7	F	Trip over running	8	53°	34%	-	-	-
14	6	F	Fall onto arm	7	76°	49%	-	-	-
15	7	M	Fall playing soccer	14	23°	36%	-	-	-

**Table 2:** Patient-level decision making for proceeding to open reduction.

Patient	Days to Surgery	Initial Fracture Angle on XR	Initial Fracture Displacement on XR	First Step: 3x Closed Reduction Attempts	Second Step: Percutaneous k-Wire Leverage Reduction	Third Step: Percutaneous Kapandji k-Wire Reduction	Outcome	Decision for Open Reduction	Intraoperative Block to Reduction
1	1	40°	32%	✓	✓	✓	Inadequate CR	✓	Annular ligament
2	10	41°	57%	✓	✓	✕ (fracture not mobile)	Minimal fracture mobilization	✓	Early callus formation
3	1	25°	20%	✓	✓	✓	Incongruent RCJ	✓	Annular ligament
4	13	21°	41%	✓	✓	✕ (fracture not mobile)	Minimal fracture mobilization	✓	Early callus formation
5	6	45°	38%	✓	✓	✓	Inadequate CR	✓	Periosteum
6	4	51°	51%	✓	✓	✓	Inadequate CR	✓	Annular ligament
7	3	63°	45%	✓	✓	✓	Inadequate CR	✓	Annular ligament
8	4	47°	50%	✓	✓	✓	Inadequate CR	✓	Annular ligament
9	9	18°	48%	✓	✓	✓	Inadequate CR	✓	Early callus formation
10	4	22°	52%	✓	✓	✓	Inadequate CR	✓	Periosteum
11	1	92°	100%	✓	✓	✓	Inadequate CR, RCJ instability	✓	Periosteum + annular ligament
12	7	37°	30%	✓	✓	✓	Incongruent and unstable RCJ	✓	Annular ligament
13	8	53°	34%	✓	✓	✓	Inadequate CR	✓	Periosteum + early callus formation
14	7	76°	49%	✓	✓	✓	Inadequate CR	✓	Periosteum + annular ligament
15	14	23°	36%	✓	✓	✕ (fracture not mobile)	Minimal fracture mobilization	✓	Early callus formation

RCJ = radiocapitellar joint. CR = closed reduction. ✓ = performed. ✕ = not performed.

**Table 3:** Patient outcome data.

Patient	Duration of Follow-Up (in Months)	Flexion	Extension	Supination	Pronation	Union	Time to Union (in Weeks)	AVN	Radial Head Subluxation	Subsequent Procedures	Subsequent Procedure Timeframe (in Months)	Other Complications
1	12.9	140°	0°	90°	90°	Yes	11.0	No	No	Removal of metal	12.6	Mild HO
2	6.0	145°	0°	90°	90°	Yes	4.6	No	No	Removal of metal	2.9	-
3	8.7	145°	−5°	80°	80°	Yes	13.4	No	No	Removal of metal	5.8	Mild HO
4	19.9	145°	−10°	60°	90°	Yes	4.3	No	No	Removal of metal	6.2	-
5	15.1	135°	5°	60°	70°	Yes	17.0	No	No	Removal of metal	4.8	Physeal closure
6	17.3	145°	0°	70°	90°	Yes	5.5	No	No	Removal of metal	3.9	-
7	14.1	145°	0°	45°	90°	Yes	7.4	No	No	Removal of metal, Arthroscopic PRUJ release including ulna ostectomy	6.0	-.
8	9.0	145°	0°	80°	90°	Yes	10.8	No	No	Planned removal of metal		-
9	4.5	140°	5°	90°	90°	Yes	3.9	No	No	Removal of metal	1.6	-
10	8.1	135°	5°	50°	70°	Yes	12.7	No	No	Removal of metal	5.8	-
11	8.6	110°	20°	40°	60°	Yes	18.9	Yes	No	Revision of fixation as elastic nail migrated into joint, revised to plate with ulna bone graft	2.1	Migratory metalware which became intra-articular at 8 weeks post-surgery
12	8.0	140°	−5°	90°	90°	Yes	4.9	No	No	Removal of metal	8.0	-
13	5.7	140°	−5°	30°	80°	Yes	12.7	No	No	Planned removal of metal, arthroscopic debridement of scar tissue		-
14	5.7	140°	5°	90°	90°	Yes	6.9	No	No	Removal of metal	5.7	-
15	4.9	130°	0°	30°	10°	Yes	5.3	No	No	Removal of metal	4.9	HO with limited pronosupination
Mean	9.9	139°	1°	66°	79°		9.3				5.4	
Standard Deviation	4.8	9°	7°	22°	21°		4.9				2.8	

**Table 4:** Isolated radial neck injury vs. radial neck fracture with associated injury outcome data. *n* = number.

		Age	Follow-Up Duration (Months)	Days to Surgery	Flexion	Extension	Supination	Pronation	Initial Fracture Angle on XR	Initial Fracture Displacement on XR	Time to Union (in Weeks)
Isolated Injuries (*n* = 8)	Mean	7.7	9.9	7.5	140°	−1°	66°	75°	41°	38%	9.4
	SD	2.0	5.5	4.9	5.3°	5°	26°	27°	18°	11%	4.8
Associated Injuries (*n* = 7)	Mean	7.8	9.9	4.6	137°	4°	66°	83°	47°	54%	9.2
	SD	2.4	4.3	2.6	13°	8°	21°	13°	25°	22%	5.4

## Data Availability

The original contributions presented in this study are included in the article. Further inquiries can be directed to the corresponding author.

## Abbreviations

The following abbreviations are used in this manuscript:

AVN	Avascular Necrosis
SD	Standard Deviation
CI	Confidence Interval
ESIN	Elastic Stable Intramedullary Nail
LUCL	Lateral Ulnar Collateral Ligament
ECRB	Extensor Carpi Radialis Brevis
EDC	Extensor Digitorum Communis
PIN	Posterior Interosseous Nerve
HO	Heterotopic Ossification
RCJ	Radiocapitellar Joint
CR	Closed Reduction
PRUJ	Proximal radioulnar joint
